# Supplementary material for: Virucidal and Antibacterial Chitosan–NanoCu Film-Coating-Based Technology: Complete Analysis of Its Performance on Various Surfaces
Source: Viruses. 2025 Oct 7;17(10):1347. doi: 10.3390/v17101347 (PMC12567648; doi:10.3390/v17101347)
Supplement: Supplementary file 1 [file viruses-17-01347-s001.zip › viruses-3879182-Table S1.pdf]

Supplementary Table S1. Characteristics of the viruses employed.

**Virus**	**Envelope**	**Genome**	**Incubation Times (Days)**
HSV-1	Enveloped	DNA	2
HSV-1 tk^-	Enveloped	DNA	2
HSV-2	Enveloped	DNA	2
RSV	Enveloped	RNA	5
BCoV	Enveloped	RNA	3
ZIKV	Enveloped	RNA	5
ADV	Non-enveloped	DNA	7
PV-1	Non-enveloped	RNA	2
