# Supplementary figures and images for: Virucidal and Antibacterial Chitosan–NanoCu Film-Coating-Based Technology: Complete Analysis of Its Performance on Various Surfaces
Source: Viruses. 2025 Oct 7;17(10):1347. doi: 10.3390/v17101347 (PMC12567648; doi:10.3390/v17101347)

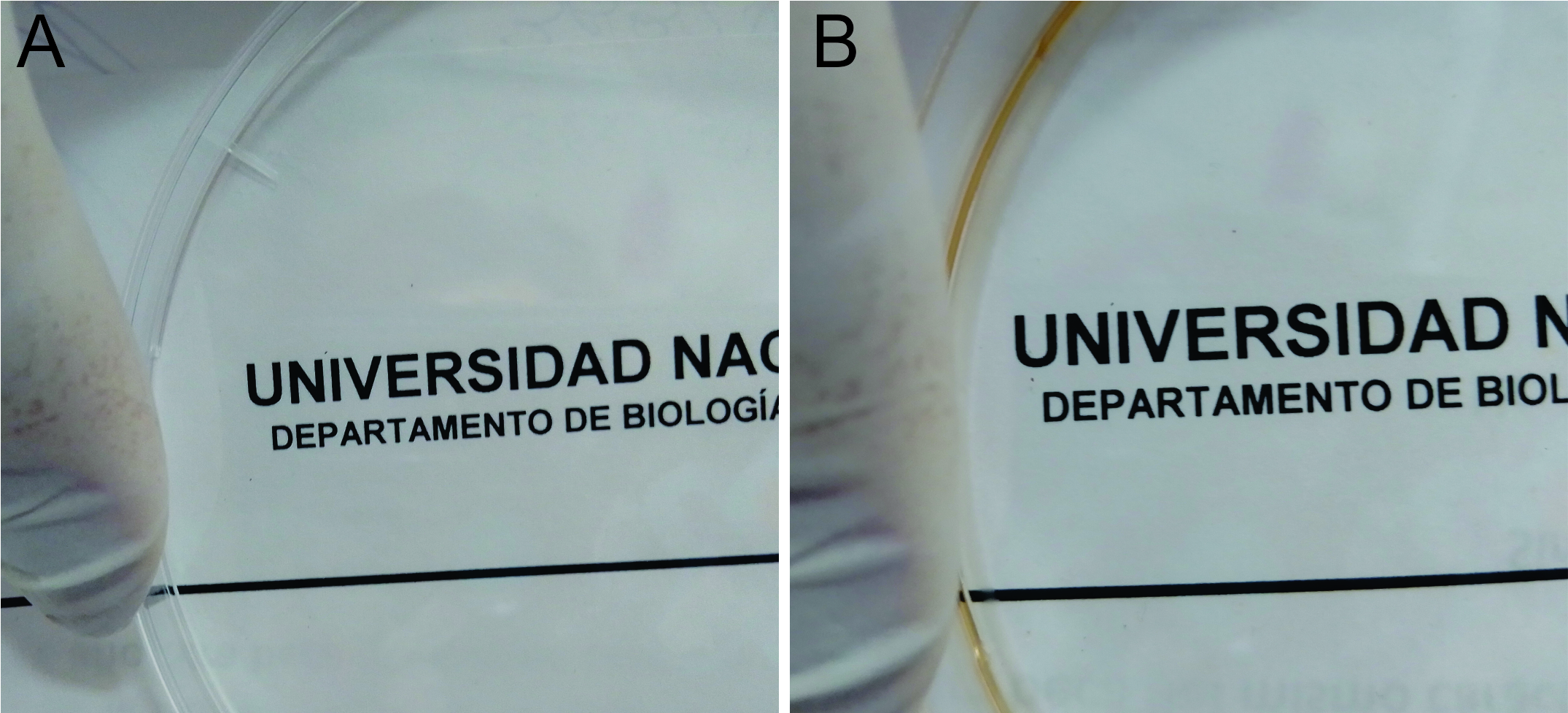

Supplement: Supplementary file 1 [file viruses-17-01347-s001.zip › viruses-3879182-Figure S1.tif]
